# Informing thresholds for paediatric transfusion in Africa: the need for a trial

**DOI:** 10.12688/wellcomeopenres.15003.2

**Published:** 2019-08-12

**Authors:** Kathryn Maitland, Eric O. Ohuma, Ayub Mpoya, Sophie Uyoga, Oliver Hassall, Thomas N. Williams

**Affiliations:** 1 Department of Medicine, Imperial College London, London, W2 1PG, UK; 2 Clinical Trials Facility, KEMRI-Wellcome Trust Research Programme, Kilifi, PO Box 230, Kenya; 3 Nuffield Department of Medicine, Oxford University, Oxford, OX3 7BN, UK; 4 Epidemiology and Demographic Surveillance, KEMRI Wellcome Trust Research Programme, Kilifi, PO Box 230, Kenya; 5 Department of International Public Health, Liverpool School of Tropical Medicine, Liverpool, L3 5QA, UK

**Keywords:** Severe Anaemia, Children, Kenya, Africa, Mortality, Transfusion, Sepsis, Malaria, Guidelines

## Abstract

**Background**: Owing to inadequate supplies of donor blood for transfusion in sub-Saharan Africa (sSA) World Health Organization paediatric guidelines recommend restrictive transfusion practices, based on expert opinion. We examined whether survival amongst hospitalised children by admission haemoglobin and whether this was influenced by malaria infection and/or transfusion.

**Methods**: A retrospective analysis of standardised clinical digital records in an unselected population of children admitted to a rural hospital in Kenya over an 8-year period. We describe baseline parameters with respect to categories of anaemia and outcome (in-hospital death) by haemoglobin (Hb), malaria and transfusion status.

**Results**: Among 29,226 children, 1,143 (3.9%) had profound anaemia (Hb <4g/dl) and 3,469 (11.9%) had severe anaemia (Hb 4-6g/d). In-hospital mortality rate was 97/1,143 (8.5%) if Hb<4g/dl or 164/2,326 (7.1%) in those with severe anaemia (Hb ≥4.0-<6g/dl). Admission Hb <3g/dl was associated with higher risk of death versus those with higher Hbs (OR=2.41 (95%CI: 1.8 - 3.24; P<0.001), increasing to OR=6.36, (95%CI: 4.21–9.62; P<0.001) in malaria positive children. Conversely, mortality in non-malaria admissions was unrelated to Hb level. Transfusion was associated with a non-significant improvement in outcome if Hb<3g/dl (malaria-only) OR 0.72 (95%CI 0.29 - 1.78), albeit the number of cases were too few to show a statistical difference. For those with Hb levels above 4g/dl, mortality was significantly higher in those receiving a transfusion compared to the non-transfused group. For non-malarial cases, transfusion did not affect survival-status, irrespective of baseline Hb level compared to children who were not transfused at higher Hb levels.

**Conclusion**: Although severe anaemia is common among children admitted to hospital in sSA (~16%), our data do not indicate that outcome is improved by transfusion irrespective of malaria status. Given the limitations of observational studies, clinical trials investigating the role of transfusion in outcomes in children with severe anaemia are warranted.

## Abbreviations

Hb Haemoglobin; KCH Kilifi County Hospital; mps malaria parasites; MUAC mid-upper arm circumference; RCT randomised controlled trial; OR Odds ratios; rbc red blood cells; sSA sub-Saharan Africa; wbc white blood cells; WHZ Weight for Height Z score; WHO World Health Organization.

## Introduction

Transfusion of blood can be a life-saving intervention, and provision of adequate supplies of safe blood for transfusion are an essential undertaking for any health system. Issues of blood safety, adequate supply, equitable access and rational use, however, remain key challenges throughout the world. In resource-limited countries in sub-Saharan Africa (sSA) these issues present major barriers to the development of successful nationally-coordinated blood transfusion services ^1^. The World Health Organization (WHO) Global Database on Blood Safety reports a mismatch between supply and demand. For example, the 2016 WHO survey found that of the 46 countries reporting from the WHO African Region, (which are home to approximately 13% of the global population) overall they collected a total of approximately 5.6 million blood donations, which accounted for only 4% of global donations ^2^. In an earlier report the average blood donation rate was 2.3/1000 population in countries with a low human development index in comparison to 36.7 in countries with a high human developmental index (HDI) ^2, 3^. The figure for the Africa Region (excluding South Africa) is only 3.4/1000 compared to the WHO estimated optimal requirement of 10-20/1000 ^4^.

In order to bridge the major gap between supply and demand, one of the four key goals, mandated in a WHO resolution on an integrated strategy of blood safety in 1975 was to ‘reduce unnecessary transfusions’ through the more effective clinical use of blood and the use of simple alternatives to transfusion (such as crystalloids and colloids) where possible ^5^. WHO has subsequently developed and published guidelines for the appropriate use of blood for patient groups suffering the greatest supply shortages ^6, 7^. Notably, the pattern of blood utilisation in sSA is very different from that in high HDI nations, where elective-use predominates and where supply is strictly monitored through specialist transfusion services. By contrast, the 2016 WHO survey on world safety and availability of blood transfusions found that in low-and-middle-income countries, 67% of transfusions are received by children under 5 years old, followed by women for pregnancy-related complications ^2^ with most being given as emergency interventions ^8^.

### What is already known?

For paediatric transfusion, the WHO conservative policy reserves blood transfusion for children with a Hb <4g/dl or for those with an Hb <6g/dl if accompanied by life-threatening complications ^6, 7^. These specific recommendations have not been systematically evaluated. Consequently, compliance is often poor and many children receive unwarranted transfusions ^9, 10^. Nevertheless, adverse outcomes following admission to hospital with severe anaemia in children are common, with case-fatality rates being high both within-hospital (9–10%) ^11^ and within 6-months of discharge (12%) ^12^, in common with rates of relapse or re-hospitalisation (6%) ^12^. Such data suggest that current recommendations and management strategies may not be working in practice. Although the conservative WHO transfusion guidelines were developed to protect scarce resources, little research has since been conducted to either support or challenge the haemoglobin thresholds for administering a transfusion. With this in mind, we have conducted a retrospective analysis of mortality outcome by Hb level at admission in an unselected paediatric population admitted to a rural district hospital in Kenya over an 8-year period. Secondary aims were to examine whether outcome (survival) was influenced by malaria infection and/or transfusion.

## Methods

The study was conducted on the paediatric wards at Kilifi County Hospital (KCH), a rural district hospital on the coast of Kenya, where a system of routine surveillance has been operated by the KEMRI-Wellcome Trust Research Programme since 1989. All children <15 years of age on admission to KCH were assessed by a clinician. Standardised clinical proforma are entered directly onto a computerized database. In addition, all patients were investigated with a standard set of laboratory tests including a full blood count, a blood culture and a blood film for malarial parasites. HIV status was tested following parental assent. At discharge (or in fatal cases) the clinician completed a standard summary onto the computerized database which included discharge diagnosis and whether a blood transfusion had been given. Blood transfusion policy at KCH follows WHO guidelines.

All admission records for the 8-year period January 2002 to September 2009 were included in the current analysis. Throughout this period a single method for Hb measurement (Coulter counter, Coulter Electronics) was used thus minimising potential methodological variation. Clinical data on key variables were retrieved together with the key co-morbidities, including bacteraemia, malarial parasitaemia and nutritional and HIV status, the receipt of a whole blood transfusion during admission and discharge status (alive or dead).

### Data analysis

**Figure 1.**
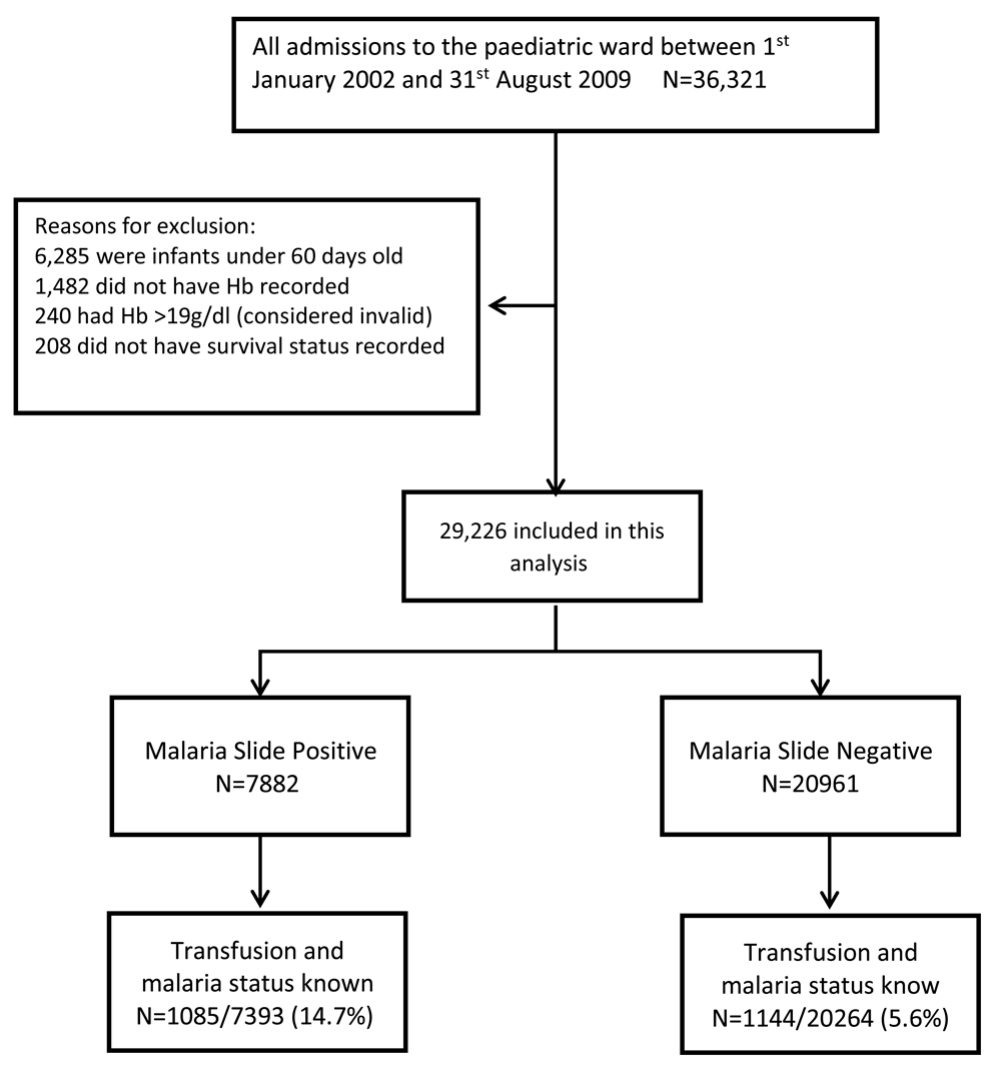
Study flow.

Our analysis included data from 36,621 consecutive admissions to the KCH paediatric ward (See Figure 1: Study Flow). Patients with missing data on the primary exposure (admission Hb); 1,482 observations, 4.0%) or on the primary outcome (in-hospital mortality as defined by status at discharge; 208 observations, 0.1%) were excluded from the analysis. Infants under 60 days (6,285 observations, 17%) and cases with a Hb well outside the normal range, >19.0 g/ld. (240, 0.1%) were excluded. With these adjustments, 29,226 patients remained for analysis. Z-scores for the anthropometric parameters weight-for-age (WAZ) were calculated for each individual using Epi Info v2000 (CDC, Atlanta) and undernutrition defined as a WAZ of <-3 while severe malnutrition was defined as a mid-upper arm circumference (MUAC) of <11.5cm. Dichotomous and categorical variables were created from continuous variables. Shock was defined on the basis of one or more of the following clinical features: capillary refill time >2 seconds; weak pulse or a temperature gradient in the lower limbs (defined as a temperature cline detected when running the back of the hand down from thigh to shin). We also examined whether the clinicians were compliant with WHO guidelines for transfusion. Compliance was considered using only the features from admission. The WHO restricts transfusion to those with Hb <4g/dl or for those with an Hb <6g/dl if accompanied by life-threatening complications or severity features (any one of shock (any feature of delayed capillary refilling time more than 2 seconds, weak pulse or cold extremities (temperature gradient, severe dehydration (sunken eyes or decreased skin turgor), impaired consciousness (prostration or coma (inability to localise a painful stimulus), respiratory distress or high parasitaemia at admission).

Odds ratios (OR) for mortality in patients with an Hb below (exposed group) versus those with an Hb equal to or above (unexposed) specific thresholds, determined depending on the presence or absence of additional clinical features, were assessed using logistic regression and adjusted for malaria and transfusion status to examine the risk of in-hospital death by level of Hb at admission. All analyses were conducted using STATA Version 11 (Stata Corporation, Texas, USA).

## Results

A total of 29,226 patients were included for analysis. Baseline characteristics are described by age group ( Table 1) and by the severity of anaemia ( Table 2). At admission, 1,143 (3.9%) had profound anaemia (Hb <4g/dl); 2,326 (7.1%) had severe anaemia (Hb 4- < 6.0 g/dl) and almost one third (9,457) had moderate anaemia (6.0-8.9 g/dl). The admission cohort included 5,914 (20.2%) children with malnutrition described by weight for age <-3; alternatively 4,175/28,734 (14.6%) by mid-upper arm circumference (MUAC) <11.5 cm. The majority of children (58.8%) were febrile (axillary temperature >37.%C: measured by digital thermometer) at presentation. Clinical pallor defined by examination of conjunctiva, nail bed and palms of hands or soles of feet was present in 95% of children with profound anaemia (Hb<4g/dl); 77% with Hb 4-6g/dl, and 19% of children Hb>6g/dl ( Table 2); jaundice was uncommon (1.9%).

**Table 1.** Baseline variables by age group.

	>2 to <12 months (n=8624)		12 to <24 months (n=7189)		24 to <60 months (n=8637)		>= 60 months (n=4776)		TOTAL (n=29226)	
	Freq. (%)	Died (%)	Freq. (%)	Died (%)	Freq. (%)	Died (%)	Freq. (%)	Died (%)	Freq. (%)	Died (%)
Discharge status										
Alive	8121 (94.1)	---	6847 (95.2)	---	8194 (94.9)	---	4516(94.5)	---	27678 (94.7)	---
Dead	503 (5.9)	---	342 (4.8)	---	443 (5.1)	---	260 (5.5)	---	1548 (5.3)	---
Hb at admission										
<4.0 g/ld.	254 (3.0)	20 (7.9)	285 (4.0)	21 (7.4)	381 (4.1)	39 (10.2)	223 (4.7)	17 (7.6)	1143 (3.9)	97 (8.5)
4.0 to <6.0 g/ld.	596 (6.9)	35 (5.9)	648 (9.0)	31 (4.8)	766 (8.9)	56 (7.3)	316 (6.6)	42 (13.3)	2326 (8.0)	164 (7.1)
6.0 to <9.0 g/ld.	2880 (33.4)	221 (7.7)	2701 (37.6)	161 (6.0)	2781 (32.2)	175 (6.3)	1095 (22.9)	70 (6.4)	9457 (32.4)	627 (6.6)
9.0 to <12.0 g/ld.	4593 (53.3)	199 (4.3)	3379 (47.0)	118 (3.5)	4092 (47.4)	146 (3.6)	2300 (48.2)	86 (3.7)	14364 (49.1)	549 (3.8)
>= 12.0 g/ld.	301 (3.5)	28 (9.3)	176 (2.4)	11 (6.3)	617 (7.1)	27 (4.4)	842 (17.6)	45 (5.3)	1936 (6.6)	111 (5.7)
Sex										
Female	3723 (43.2)	247 (6.6)	3166 (44.0)	160 (5.1)	3849 (44.6)	198 (5.1)	2120 (44.4)	112 (5.3)	12858 (44.0)	717 (5.6)
Male	4901 (56.8)	256 (5.2)	4023 (56.0)	182 (4.5)	4788 (55.4)	245 (5.1)	2656 (55.6)	148 (5.6)	16368 (56.0)	831 (5.1)
WAZ score <-3										
No	7360 (85.3)	297 (4.0)	5112 (71.1)	93 (1.8)	6628 (76.7)	192 (2.9)	3996 (83.7)	168 (4.2)	23096 (79.0)	750 (3.2)
Yes	1237 (14.3)	199 (16.1)	2049 (28.5)	242 (11.8)	1954 (22.6)	237 (12.1)	674 (14.1)	82 (12.2)	5914 (20.2)	760 (12.9)
Missing value	27 (0.3)	---	28 (0.4)	---	55 (0.6)	---	106 (2.2)	---	216 (0.7)	---
MUAC (<11.5 cm)										
No	6358 (73.7)	192 (3.0)	5953 (82.8)	137 (2.3)	7746 (89.7)	263 (3.4)	4482 (93.8)	200 (4.5)	24539 (84.0)	792 (3.2)
Yes	2142 (24.8)	266 (12.4)	1150 (16.0)	189 (16.4)	721 (8.4)	131 (18.2)	182 (3.8)	37 (20.3)	4195 (14.4)	623 (14.9)
Missing value	124 (1.4)	---	86 (1.2)	---	170 (2.0)	---	112 (2.4)	---	492 (1.7)	---
Febrile (>37.5°C)										
No	3403 (39.5)	97 (2.9)	2861 (39.8)	113 (3.9)	3374 (39.1)	101 (3.0)	2350 (49.2)	61 (2.6)	11988 (41.0)	372 (3.1)
Yes	5209 (60.4)	405 (7.8)	4313 (60.0)	228 (5.3)	5248 (60.8)	339 (6.5)	2412 (50.5)	198 (8.2)	17182 (58.8)	1170 (6.8)
Missing value	12 (0.1)	---	15 (0.2)	---	15 (0.2)	---	14 (0.3)	---	56 (0.2)	---
Deep breathing										
No	7362 (85.4)	316 (4.3)	6400 (89.0)	228 (3.6)	7798 (90.3)	305 (3.9)	4516 (94.6)	204 (4.5)	26076 (89.2)	1053 (4.0)
Yes	1245 (14.4)	185 (14.9)	780 (10.9)	113 (14.5)	827 (9.6)	135 (16.3)	252 (5.3)	54 (21.4)	3104 (10.6)	487 (15.7)
Missing value	17 (0.2)	---	9 (0.1)	---	12 (0.1)	---	8 (0.2)	---	46 (0.2)	---
Indrawing										
No	4890 (56.7)	214 (4.4)	5474 (76.1)	240 (4.4)	7262 (84.1)	324 (4.2)	4219 (88.3)	188 (4.5)	21845 (74.8)	966 (4.4)
Yes	3724 (43.2)	287 (7.7)	1705 (23.7)	101 (5.9)	1359 (15.7)	116 (8.5)	550 (11.5)	71 (12.9)	7338 (25.1)	575 (7.8)
Missing value	10 (0.1)	---	10 (0.1)	---	16 (0.2)	---	7 (0.2)	---	43 (0.2)	---
Delayed CRF (>=3 s)										
No	8030 (93.1)	369 (4.6)	6758 (94.0)	260 (3.8)	8101 (93.8)	325 (4.0)	4548 (95.3)	200 (4.4)	27437(93.9)	1154 (4.2)
Yes	579 (6.7)	130 (22.5)	416 (5.8)	81 (19.5)	515 (6.0)	112 (21.7)	209 (4.4)	54 (25.8)	1719 (5.9)	377 (21.9)
Missing value	15 (0.2)	---	15 (0.2)	---	21 (0.2)	---	19 (0.4)	---	70 (0.2)	---
WHO shock definition										
No	7119 (82.6)	300 (4.2)	5931 (82.5)	199 (3.4)	7125 (82.5)	264 (3.7)	4125 (86.4)	164 (4.0)	24300 (83.2)	927 (3.8)
Yes	1500 (17.4)	202 (13.4)	1246 (17.3)	142 (11.4)	1498 (17.3)	176 (11.7)	635 (13.3)	92 (14.5)	4879 (16.7)	612 (12.5)
Missing value	5 (0.1)	---	12 (0.2)	---	14 (0.2)	---	16 (0.3)	---	47 (0.2)	---
Coma (BCS <= 2)										
No	8258 (95.8)	387 (4.7)	6856 (95.4)	281 (4.1)	7904 (91.5)	295 (3.7)	4517 (94.6)	204 (4.5)	27535 (94.2)	1167 (4.2)
Yes	351 (4.1)	114 (32.5)	321 (4.5)	59 (18.4)	720 (8.3)	145 (20.1)	244 (5.1)	55 (22.5)	1636 (5.6)	373 (22.8)
Missing value	15 (0.2)	---	12 (0.2)	---	13 (0.2)	---	15 (0.3)	---	55 (0.2)	---
Bacteraemia										
No	7968 (92.4)	383 (4.8)	6656 (92.6)	285 (4.3)	7993 (92.5)	362 (4.5)	4139 (86.7)	197 (4.8)	26756 (91.6)	1227 (4.6)
Yes	422 (4.)	106 (25.1)	291 (4.1)	55 (18.9)	296 (3.4)	70 (23.6)	249 (5.2)	51 (20.5)	1258 (4.3)	282 (22.4)
Missing value	234 (2.7)	---	242 (3.4)	---	348 (4.0)	---	388 (8.1)	---	1212 (4.1)	---
Malaria Parasites										
No	7304 (84.7)	460 (6.3)	5207 (72.4)	275 (5.3)	5043 (58.4)	294 (5.8)	3521 (73.7)	221 (6.3)	21075 (72.1)	1250 (5.9)
Yes	1247 (14.5)	37 (3.0)	1920 (26.7)	62 (3.2)	3526 (40.8)	145 (4.1)	1202 (25.2)	36 (3.0)	7895 (27.0)	280 (3.5)
Missing value	73 (0.8)	---	62 (0.9)	---	68 (0.8)	---	53 (1.1)	---	256 (0.9)	---
HIV positive *										
No	2050 (93)	100 (4.9)	1751 (92.7)	71 (4.1)	1881 (91.0)	63 (3.3)	1205 (90.9)	43 (3.6)	6887 (92.1)	277 (4.0)
Yes	147 (6.7)	33 (22.4)	138 (7.3)	23 (16.7)	187 (9.0)	33 (17.6)	121 (9.1)	20 (16.5)	593 (7.9)	109 (18.4)

*Excluding cases that were not tested, discordant, and missing observations (number excluded = 21746 (74.4%))

**Table 2.** Baseline characteristics by severity of anaemia.

Variable	Hb <4g/dl n (%)	Hb 4 to <6 g/dl n (%)	Hb ≥6/dl n (%)	Total n (%)
N (%)	N = 1143 (3.9)	N =2326 (8.0)	N = 25,757 (88.1)	N = 29226
Age Group (months) 2–11 m 12–23m 24–59m ≥60 m	254 (22.2) 285 (24.9) 381 (33.3) 223 (19.5)	596 (25.6) 648 (27.9) 766 (32.9) 316 (13.6)	7774 (30.2) 6256 (24.3) 7490 (29.1) 4237 (16.5)	8624 (29.5) 7189 (24.6) 8637 (29.6) 4776 (16.4)
Sex Males (%)	603 (52.8)	1282 (55.1)	14483 (56.2)	16368 (56.0)
Fever: axillary temp ≥37.5C	591 (51.8)	1454 (62.5)	15137 (58.8)	17182 (58.8)
Pallor	1086 (95.0)	1796 (77.2)	4967 (19.3)	7849 (26.9)
Visable Jaundice	55 (4.8)	124 (5.3)	370 (1.4)	549 (1.9)
WAZ ≤ -3 ^a^	354 (31.0)	667 (28.7)	4893 (19.0)	5914 (20.2)
MUAC ≤11.5 cm ^b^	213 (18.6)	427 (18.4)	3555 (13.8)	4195 (14.4)
Weak pulse volume	163 (14.3)	143 (6.2)	1051 (4.1)	1357 (4.6)
Tachycardia ^13^	115 (10.1)	412 (17.7)	3884 (15.1)	4411 (15.1)
Capillary refill time ≥3s	300 (26.3)	283 (12.2)	1136 (4.4)	1719 (5.9)
Temperature gradient	245 (21.4)	419 (18.0)	3563 (13.8)	4227 (14.5)
Strict shock definition ^c^	35 (3.1)	13 (0.6)	94 (0.4)	142 (0.5)
Severe dehydration ^d^	81 (7.1)	200 (8.6)	3967 (15.4)	4248 (14.5)
Indrawing	279 (24.4)	545 (23.4)	6514 (25.3)	7338 (25.1)
Deep breathing ^e^	252 (22.1)	350 (15.1)	2502 (9.7)	3104 (10.6)
Hypoxaemia(pulse oximetry) Oxygen saturation <90%	118 (10.3)	138 (5.9)	1232 (4.8)	1488 (5.1)
Base excess <-8 ^f^	383 (52.2)	547 (35.1)	5632 (39.9)	6562 (40.0)
Conscious level ^g^ Alert/Normal Prostration/Coma	809 (83.8) 157 (16.3)	1739 (87.1) 257 (12.9)	20998 (94.3) 1274 (5.7)	23546 (93.3) 1688 (6.7)
High malaria parasitaemia ^h^	539 (47.2)	1252 (53.8)	6104 (23.7)	7895 (27.0)
HIV antibody positive ^i^	17/223 (7.6)	52/446 (11.7)	524/6811 (7.7)	593/7480 (7.9)
Pathogenic bacterial isolate	63 (5.5)	172 (7.4)	1023 (4.0)	1258 (4.3)

^a^WAZ: weight for age Z-score ^b^MUAC: mid-upper arms circumference ^c^Presence of weak pulse volume & capillary refill time ≥3s & temperature gradient ^d^Presence of sunken eyes of decreased skin turgor ^e^Kussmaul’s breathing ^f^Excludes missing observations (for base excess: number of missing observations = 12819 (43.9%), for conscious level: number of missing observations = 3992 (13.7%) ^g^Prostration: inability to sit upright (if >8months) or feed; coma failure to localise a painful stimulas ^j^hyperparasitaemia defined as:
percentage_parasitaemia= (malaria parasites (mps) per 100 white blood cells (wbc)/100)*wbc*1000 >10, or as percentage_parasitaemia= (mps500 red blood cells (rbc)/500)*rbc*1000000 >10
^i^Excluding cases that were not tested, discordant, and missing observations (number excluded = 21746 (74.4%))

Deep ‘acidotic’ breathing and/or indrawing (respiratory distress) were present in 3,104/29,180 (10.6%) and 7,338/29,183 (25.1%), respectively. Respiratory distress was associated with higher case fatalities 487/3104 (15.7%) and 575/7,338 (7.8%), respectively, compared to 1053/26,076 (4.0%) or 966/21,845 (4.4%) in children without either of these signs. Notably few had hypoxaemia (defined as a pulse oximetry (pSO _2_) reading of less that 90%) present in on 118/1143 (10.3%) in children with profound anaemia. Clinically-defined shock was present in 4,879 (16.7%) and was associated with a higher fatality 612 (12.5%) compared to those without shock 927/24,300 (3.8%). Overall, 1,249/27909 (4.5%) children had culture-proven bacteraemia, with a case fatality exceeding 20% ( Table 1). In children with profound anaemia and severe anaemia 47% and 53% were malaria-parasite positive; however overall mortality was lower, relative to other co-morbidities with 280 deaths (case fatality rate, 3.6%). HIV status (antibody) data are less reliable owing to the large number of missing data (17,974 (61.5%)).

### Compliance with WHO guidelines

Overall, compliance with WHO transfusion guidelines was good, especially with respect to transfusions given to children with an admission Hb >6g/dl, which occurred in only 520/24372 (2.1%). For those with Hb< 4gdl (n=1134), 912 (80%) were transfused ( Table 3). Children with an admission Hb 4-6g/dl, which included 1629/27,904 (5.8%) of children at hospital admission, 1564 (5.6%) of all admissions met the severity criteria to transfuse, but only 567 (36.3%) were transfused and 746 did not met severity criteria yet 194 (26% of this category) were transfused ( Table 3). The reasons for transfusion or no transfusion were not systematically captured on the database.

**Table 3.** Receipt of transfusion according to World Health Organization transfusion (WHO) criteria.

WHO Criteria	Category	Freq. (%)	Transfused (%)	Numbers transfused in sub-category (%)
Eligible for transfusion	Hb < 4	1134 (4.1)	912 (80.4)	1479 (54.8)
	Hb 4-6: Criteria +ve	1564 (5.6)	567 (36.3)	
Ineligible for transfusion	Hb 4-6: Criteria -ve	746 (2.7)	194 (26.0)	714 (2.8)
	Hb > 6	24372 (87.3)	520 (2.1)	

Criteria +ve includes any one of   •   Clinically detectable dehydration   •   Shock (compensated)   •   Impaired consciousness   •   Deep breathing   •   Very high parasitaemia Criteria – ve includes none of the above

### Mortality by severity of anaemia

Profound anaemia (Hb<4g/dl) was associated with the highest fatality 97/1,143(8.5%) compared to 164/2,326 (7.1%) with severe anaemia (Hb ≥4.0-<6g/dl); 627/9,457 (6.6%) with moderate anaemia (≥6.0-<9.0 g/dl) and 660/16,300 (4.0%) without anaemia (Hb≥9g/dl) ( Table 1). The increased risk of mortality in comparison to children with a higher admission Hb level was greatest (OR=1.70; 95%CI: 1.37-2.11) among children with profound anaemia (Hb<4g/dl). The strength of this association was less apparent when comparing the probability of death across the anaemia subgroups, for example profound anaemia vs severe anaemia (P=0.13) and in severe anaemia vs moderate anaemia (P=0.80).

**Figure 2.**
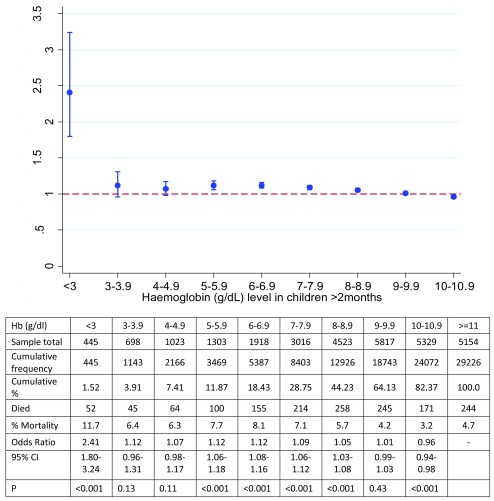
Odds of mortality by haemoglobin (Hb) level status.

We therefore conducted an in-depth examination of the risk of fatal outcome for each stratum of Hb level compared to all Hb’s above that stratum in an attempt to validate the current haemoglobin thresholds for transfusion ( Figure 2). Overall, children with a Hb of <3g/dl were at significantly higher risk of death compared to those with a higher admission Hb (OR= 2.41 (95%CI: 1.8 - 3.24; P<0.001). Conversely, the risk of death with a Hb 3-3.9g/dl compared to those with a higher Hb was less pronounced OR=1.12 (95%CI: 0.96 - 1.31; P=0.13). Similarly, children who fall within the classification of severe or moderately-severe anaemia had very little variation in their risk of mortality across the whole range of haemoglobins from 4.0 to 9.9g/dl.

### Outcome in relation to malaria

**Figure 3.**
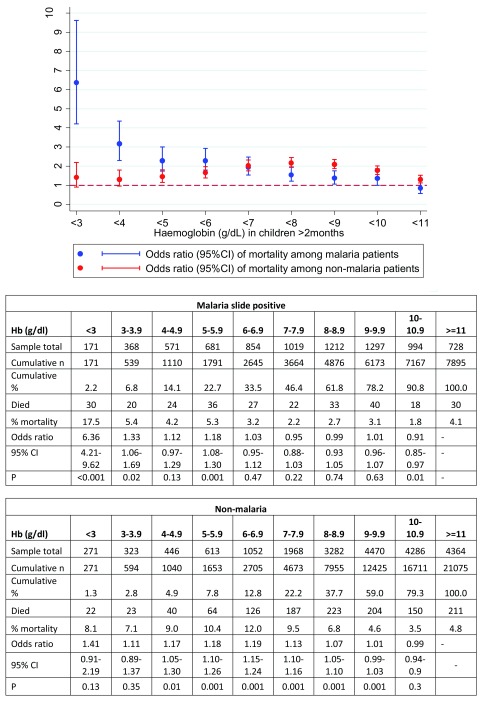
Odds of mortality by haemoglobin (Hb) level among those with *Plasmodium falciparum* malaria (blue) and those without (red). Odds ratios compared risk of mortality at each Hb level to that above that specific threshold.

We analysed the risk of mortality separately for children with *Plasmodium falciparum* malaria versus non-malarial (blood slide-negative) admissions ( Table 4a, Table 4b and Figure 3). There were substantial differences in the patterns of risk by haemoglobin level. Among children with malaria parasitaemia, the greatest risk of death was at Hb’s of <3g/dl (OR=6.36, 95%CI: 4.21–9.62) compared to children with higher Hb’s, whereas the risk was less marked at an Hb level of 3-3.9g/dl compared to children with higher haemoglobins (OR=1.33, 95%CI: 1.06–1.69; P=0.02). Mortality was only marginally increased across Hb values between 4 and 5.9.

**Table 4a.** Children with malaria: Odds of death at each level of haemoglobin (Hb) in those who received transfusion versus no transfusion.

Hb level	N	Died	Not transfusion	Deaths non- transfused	Case fatality	Transfused	Deaths transfused	Case fatality	Odds ratio (95% CI)	P value
**<3**	169	29	38	8	21.1%	131	21	16.0%	0.72 (0.29 - 1.78)	0.471
**3-3.9**	366	20	62	5	8.1%	304	15	4.9%	0.59 (0.21 - 1.69)	0.328
**4-4.9**	552	24	250	5	2.0%	302	19	6.3%	3.29 (1.21 - 8.94)	0.02
**5-5.9**	657	36	479	16	3.3%	178	20	11.2%	3.66 (1.85 - 7.24)	<0.001
**6-6.9**	795	25	721	16	2.2%	74	9	12.2%	6.10 (2.59 - 14.35)	<0.001
**7-7.9**	948	20	911	17	1.9%	37	3	8.1%	4.64 (1.30 - 16.59)	0.018
**8-8.9**	1123	31	1,099	29	2.6%	24	2	8.3%	3.35 (0.75 - 14.94)	0.112
**9-9.9**	1186	34	1,169	28	2.4%	17	6	35.3%	22.23 (7.68 - 64.35)	<0.001
**10-10.9**	941	15	930	15	1.6%	11	0	0.00%	-	
**>=11**	656	22	649	21	3.2%	7	1	14.3%	4.98 (0.57 - 43.27)	0.145
**Total**	7393	256	6308	160	2.5%	1085	96	8.8%		

**CI confidence interval**

**Table 4b.** Children without malaria: Odds of death at each level of haemoglobin (Hb) in those who received transfusion versus no transfusion.

Hb level g/dl	N	Died	Not transfusion	Deaths non- transfused	Case fatality	Transfused	Deaths transfused	Case fatality	Odds ratio (95% CI)	P value
**<3**	266	20	54	4	7.4%	212	16	7.55%	1.02 (0.33 - 3.19)	0.972
**3-3.9**	323	23	64	5	7.8%	259	18	6.95%	0.88 (0.31 - 2.47)	0.81
**4-4.9**	436	40	214	15	7.0%	222	25	11.26%	1.68 (0.86 - 3.29)	0.127
**5-5.9**	586	61	483	34	7.0%	103	27	26.21%	4.69 (2.68 - 8.22)	<0.001
**6-6.9**	1013	121	931	85	9.1%	82	36	43.90%	7.79 (4.77 - 12.71)	<0.001
**7-7.9**	1884	180	1,815	142	7.8%	69	38	55.07%	14.44 (8.72 - 23.91)	<0.001
**8-8.9**	3154	209	3,090	173	5.6%	64	36	56.25%	21.68 (12.93 - 36.36)	<0.001
**9-9.9**	4316	192	4,267	169	3.96%	49	23	46.94%	21.45 (11.99 - 38.38)	<0.001
**10-10.9**	4115	142	4,074	129	3.2%	41	13	31.71%	14.20 (7.19 - 28.05)	<0.001
**>=11**	4171	193	4,128	174	4.2%	43	19	44.19%	17.99 (9.67 - 33.47)	<0.001
**Total**	20264	1181	19120	930	4.9%	1144	251	21.9%		

**Diagnoses for those with Hb 8.0g and above (tables 3a and b) who were transfused:** Congenital abnormality (5); cerebral palsy (3); gastroenteritis (18); hepatitis (3), HIV (22); hypersplenism (1); Lower respiratory tract infection (18); malaria (45); malignancies (15); malnutrition (56); poisoning (5); renal failure (7); sickle cell disease (14); sepsis (23); snake bite (1); surgical (7); trauma (5) and unknown (6).

The picture was different in those without malaria parasites in which risk of mortality was not clearly related to the severity of anaemia - mortality in those with Hb <3g/dl was 22/271 (8.1%) rising to 126/1052 (12%) in those with Hb 6.0-6.9 g/dl. The risk of mortality was greatest in those with Hb levels between 5-9.9g/dl.

### Effect of transfusion on outcome

We then investigated whether mortality risk was affected by the receipt of a transfusion during admission ( Table 4a & Table 4b). In those with malaria ( Table 4a), compared children who were not transfused, receipt of a transfusion appeared to improve survival for those with Hb <4g/dl but the confidence intervals were very wide (and included a possibility of harm) owing to small numbers. For all Hb levels above 4g/dl our analysis indicates that mortality was significantly higher in those receiving a transfusion compared to the non-transfused group ( Table 4a).

For non-malarial cases ( Table 4b), the receipt of a transfusion did not appear to result in a survival benefit irrespective of baseline haemoglobin level compared to children who were not transfused. Children with Hb ≥8g/dl had a substantially increased risk of mortality if transfused; however, this included a heterogeneous group of children in whom the underlying disease or cause of admission was an important determinant of their outcome (foot of Table 4b).

Finally, we examined whether the addition of clinical signs of severity (deep acidotic breathing and/or altered consciousness (prostration or coma)) were useful in identifying particularly high-risk groups in whom the receipt of a transfusion may be of benefit ( Table 5). Overall this group had a much higher mortality, and whilst the receipt of a transfusion may have improved survival for those with a Hb of <5g/dl numbers were too small to show a statistical difference. For those with a Hb ≥5g/dl and signs of severity, transfusion was associated with a substantially increased risk of mortality ( Table 5).

**Table 5.** Children with signs of severity: odds of death at each level of haemoglobin (Hb) in children who received transfusion versus no transfusion.

Hb level g/dl	Total	Deaths	Not transfused	Deaths non- transfused	Case fatality	Transfused	Deaths transfused	Case fatality	Odds ratio (95% CI)	P value
**<3**	135	30	30	9	30.00%	105	21	20.0%	0.58 (0.23 - 1.46)	0.249
**3-3.9**	167	22	32	6	18.75%	135	16	11.9%	0.58 (0.21 - 1.63)	0.304
**4-4.9**	215	32	70	11	15.71%	145	21	14.5%	0.91 (0.41 - 2.01)	0.812
**5-5.9**	252	40	137	13	9.49%	115	27	23.5%	2.93 (1.43 - 5.99)	0.003
**6-6.9**	306	60	247	37	14.98%	59	23	39.0%	3.63 (1.93 - 6.80)	<0.001
**7-7.9**	410	66	377	51	13.53%	33	15	45.5%	5.33 (2.53 - 11.23)	<0.001
**8-8.9**	533	72	504	57	11.31%	29	15	51.7%	8.40 (3.86 - 18.31)	<0.001
**9-9.9**	624	71	608	63	10.36%	16	8	50.0%	8.65 (3.14 - 23.85)	<0.001
**10-10.9**	601	61	585	53	9.06%	16	8	50.0%	10.04 (3.62 - 27.83)	<0.001
**>=11**	654	93	634	83	13.09%	20	10	50.0%	6.64 (2.68 - 16.43)	<0.001
**Total**	3897	547	3224	383	11.9%	673	164	24.4%		

## Discussion

We have shown in a retrospective analysis of 29,226 unselected children admitted to hospital on the coast of Kenya that the burden of moderate and profound or severe anaemia is substantial, affecting 15.8% of all admissions over 60 days of age, and that it carries a higher risk of in-hospital mortality (6.7–7.6%) compared to children with admission Hb >9g/dl (4%). The Hb level associated with the highest case fatality was different in children with malaria (all levels below Hb 3g/dl, 17.5%) in comparison to those without malaria (maximal at Hb 6-7g/dl; 12%). Receipt of a whole blood transfusion was associated with improved survival in malarial cases but only at admission Hb levels of <4g/dl, whereas transfusion in non-malaria cases did not appear to improve survival, irrespective of haemoglobin level, although causality cannot be inferred. These data are important when considering the current WHO recommended transfusion thresholds, especially for parts of sSA where malaria has declined or is of less public health consequence and where reconsideration of the current guidelines may be warranted. Whilst our data provide support for the current recommendations and indicate that it may be too soon to amend these, in non-malarious areas there are a number of notable limitations in both the evidence-base for the current guidelines and potential biases within our findings.

Overall, our findings were that at our centre compliance with WHO transfusion guidelines was good, specifically only 2.1% of children with with admission Hb >6g/dl received a transfusion. This may have been due to the availability of repeated measures of Hb post admission to monitor children. This finding contrasts for other reports where adherence to transfusion guidelines were not followed ^9, 10^, since many transfusions initiated solely for severe pallor, a sign with poor specificity ^14^.

Earlier studies have relied on small samples and included little information on confounders, thus limiting the generalizability of the findings. Brabin and colleagues ^11^ reviewed studies reporting case fatality from malarious areas in sSA and found wide variations in outcome. The mean in-hospital case-fatality rate for severe anaemia (Hb <5 or <6g/dl depending on study definition) was 9% (range 4–39%). While mortality was significantly higher in children with a Hb <5g/dl (pooled RR=1.92 vs >5g/dl, 95% CI 1.7–2.2), evidence for an increased risk with less severe anaemia was not conclusive: although the risk of death was increased for a Hb <8g/dl, the confidence intervals were wide ^11^. The heterogeneous group of children included and outcomes observed also make it difficult to draw specific conclusions. Our study included an unselected paediatric hospital cohort and included data on clinical severity and co-morbidities. As such the cohort represents a typical paediatric population in malaria-endemic African hospitals. A further strength of our study is that it was conducted in a setting where clinicians were largely compliant with WHO transfusion guidelines. For example, for children with a Hb >6g/dl at hospital admission only 520/24372 (2.1%) received a transfusion, and overall 67% of transfusions given were ‘’appropriate’ but this was only judged from admission criteria, the number subsequently developing severe and complicated anaemia, particularly in those with Hb 4-6g/dl admission is unknown. Overall, for children who did not meet the criteria for transfusion according to the guidelines (at admission), only 2.8% (714) received a transfusion this compares very favourably with 51% at a hospital 60 km away in Mombasa, which report over a similar time period where 51% of children who received transfusion who did not met WHO guidelines ^15^. In the TRACT trial, in children with severe uncomplicated anaemia (Hb 4-6g/dl) who were not randomised to receive an immediate transfusion, close clinical and Hb monitoring for *de novo* development of signs of severity or haemoglobin drops to < 4g/dl demonstrated that 386/787 (49%) of children developed severe and complicated anaemia, 295 of these were due to drops in the Hb < 4g/dl ^16^.

### What new knowledge this study contributes

Our current analyses indicate that for children with malaria there may be a benefit of transfusion for those with profound anaemia in terms of short-term outcome (in-hospital mortality). However, for hospital admissions without malaria the receipt of a transfusion may not be beneficial irrespective of haemoglobin levels including children with profound malarial anaemia. The major limitation of these types of analyses, whilst informative about which groups to target for further evaluation of clinical practice, the nature of the design precludes any inference of causality – which can only reliability be tested in a clinical trial. For example, children with a higher admission Hb (>8g/dl) transfusion appeared to be associated with a substantially worse outcome. Exploring the final diagnoses of this group reveals that it includes a large number of children with underlying co-morbidities associated with a substantially worse outcome including malignancies, HIV infection, severe malnutrition and trauma (see Table 2b).

In current guidelines, it is recommended that wherever possible, simple alternatives to transfusion (such as crystalloids and colloids) should be used to avert unnecessary transfusion in emergencies. However, a large paediatric controlled trial of fluid resuscitation (FEAST trial) examining boluses of 20–40mls/kg of 0.9% saline and 5% human albumin in African children with shock, including 987 (32%) with severe anaemia (Hb <5g/ld.) demonstrated a 3.3% increased absolute risk of death by 48-hours (primary outcome) in the bolus-arms compared with controls (no bolus fluid strategy) ^13^. Excess mortality in bolus arms was evident in children without severe anaemia (Hb ≥5g/ld.; relative risk 1.31 (95% confidence interval 0.93-1.84)) as well as those severe anaemia (Hb <5g/ld.; RR 1.71 (1.16-2.51)) with no apparent heterogeneity between these sub-groups (p=0.31) ^13^. For the conditions studied in the trial, largely malaria and sepsis, these challenge current fluid management guidelines for children with shock but are also relevant to the recommendation for use of alternatives to transfusion.

The conservative transfusion guidelines were developed to protect scarce resources, avert overuse, and reduce the risk of transfusion-transmissible infections. However, in recent years considerable progress on strengthening transfusion services, and improving the supply and safety of transfusion through establishment of regional centres to replace hospital-based systems and by providing quality assurance for viral testing ^17^. Thus, the capacity of transfusion services to provide blood has greatly increased due to year-on-year declines in the intensity of malaria transmission that have led directly to reductions in hospitalisation of children with malaria, and indirectly to reduced utilisation of blood transfusion services ^18^. The reduction in the burden of malaria ^19^, coupled with continued poor outcomes from severe anaemia irrespective of malaria incidence, are a good starting point from which to now advocate for re-evaluation of transfusion guidelines in order to generate an evidence base for clinical practice. Such evaluation is particularly pertinent given that current recommendations were designed for areas with a high proportion of malaria-associated anaemia as opposed to severe anaemia secondary to other aetiologies where mortality appears to be much higher ^13, 20^. We have shown previously that a reduction in the transmission intensity within Kilifi District resulted in a substantial decline in malaria and paediatric admissions to the Kilifi County Hospital ^21^. Concurrent with this epidemiological transition we found a sharp decline in the prevalence of severe anaemia as well as the number and proportion of admissions transfused ^18^. Nevertheless, we found no evidence that this resulted in improved outcome, which remained constant over time despite a decrease in demand for blood on the transfusion services ^18^.

A Cochrane review including the only two African randomised controlled trials ^22, 23^ conducted to date (involving 114 and 116 children randomised to blood transfusion or oral haematinics) concluded that there was insufficient information on whether routinely giving blood to clinically stable children with severe anaemia either reduces death or results in a higher haematocrit measured at one month and indicated the need for a definitive trial ^24^. A prospective, randomised, controlled, non-inferiority trial in relatively stable Canadian and European children demonstrated that a restrictive transfusion protocol (with a transfusion threshold <7g/dl) was as safe as a liberal protocol (threshold <9g/dl) ^25^. Subsequently, practice guidelines in these countries have been amended to include restrictive transfusion (Hb<7g/dl). Worldwide consensus transfusion guidelines for stable children on intensive care units, based on the TRIPICU trial ^25^, recommend transfusion at haemoglobin <7g/dl ^26^. However they highlight the need for further trials, particularly in children with haemoglobins 5–7g/dl. Of note children in TRIPICU were likely to have had steady-state haemoglobins 11–14g/dl, conversely s African children living in areas where malaria is endemic and alpha + thalassaemia are common ^27^ typically have haemoglobin 9–11g/dl ^12, 28^. The apparent safety of not transfusion all children with lower haemoglobin in Africa may therefore reflect the differences in the steady-state haemoglobins.

## Conclusions

Despite poor compliance with current guidelines outcomes are unsatisfactory including high rates of in-hospital mortality (9–10%) and in the six months following admission with severe anaemia both case fatality and relapse remain high (6%) thus warranting a definitive trial to establish best transfusion and treatment strategies to prevent both early and delayed mortality and relapse. The Transfusion and Treatment of severe anaemia in African children: a randomised controlled trial (ISRCTN84086586) was designed to evaluate current transfusion recommendations against more liberal transfusion to improve short term and long-term outcomes to 6 months (infection prophylaxis and multi-mineral multivitamin supplementation) ^29^.

## Data availability

### Underlying data

Figshare: Admission records, including haemoglobin measurements, for 29,226 children admitted to a paediatric ward in a rural Kenyan hospital for the period 2002–2009, https://doi.org/10.6084/m9.figshare.7635908 ^30^

Data are available under the terms of the Creative Commons Zero "No rights reserved" data waiver (CC0 1.0 Public domain dedication).

### Ethics approval, consent and permissions

The Kilifi clinical surveillance was approved by Kenyan Medical Research Institute Scientific Steering Committee and National Ethics Review Committee.

Informed, written consent was obtained from parents/guardians of the research participants prior to enrolment in the surveillance studies.
