# Supplementary material for: Informing thresholds for paediatric transfusion in Africa: the need for a trial
Source: Wellcome Open Res. 2019 Aug 12;4:27. Originally published 2019 Feb 8. [Version 2] doi: 10.12688/wellcomeopenres.15003.2 (PMC6784792; doi:10.12688/wellcomeopenres.15003.2)
Supplement: Peer review history

# Peer review: Reviewer response for version 2

**DOI:** 10.21956/wellcomeopenres.16831.r36188

**Published:** 2019-10-04

**Authors:** Hans Ackerman

**Affiliations:** 1 Laboratory of Malaria and Vector Research, Division of Intramural Research, National Institute of Allergy and Infectious Diseases, National Institutes of Health, Rockville, MD, USA

**Major Comments -**

 "Our current analyses indicate that for children with malaria there may be a benefit of transfusion for those with profound anaemia in terms of short-term outcome (in-hospital mortality). However, for hospital admissions without malaria the receipt of a transfusion may not be beneficial irrespective of haemoglobin levels including children with profound malarial anaemia."

 These statements appear contradictory regarding the benefit (or not) of transfusion among children with malaria and profound anemia. Perhaps the last part of the sentence ("...including children with profound malarial anaemia.") was meant to be removed?

**Minor Comments -**

 "Overall, our findings were that at our centre compliance with WHO transfusion guidelines was good, specifically only 2.1% of children with with admission Hb >6g/dl received a transfusion."

 It may be worth balancing that statement with a comment about poor adherence among those with Hb 4-6.

**Typos, etc. - in brackets, in bold**

 "In those with malaria (Table 4a), compared **[to] **children who were not transfused, receipt of a transfusion appeared to improve survival for those with Hb <4g/dl but the confidence intervals were very wide (and included a possibility of harm) owing to small numbers."

 "For example, **[among]** children with a higher admission Hb (>8g/dl) **[,]** transfusion appeared to be associated with a substantially worse outcome."

 "However, a large paediatric controlled trial of fluid resuscitation (FEAST trial) examining boluses of 20–40mls/kg of 0.9% saline and 5% human albumin in African children with shock, including 987 (32%) with severe anaemia (Hb < **5g/ld**.) demonstrated a 3.3% increased absolute risk of death by 48-hours (primary outcome) in the bolus-arms compared with controls (no bolus fluid strategy) ^13^.  Excess mortality in bolus arms was evident in children without severe anaemia ( **Hb ≥5g/ld**.; relative risk 1.31 (95% confidence interval 0.93-1.84)) as well as those severe anaemia ( **Hb <5g/ld**.; RR 1.71 (1.16-2.51)) with no apparent heterogeneity between these sub-groups (p=0.31 ^)13^.”

I confirm that I have read this submission and believe that I have an appropriate level of expertise to confirm that it is of an acceptable scientific standard.

# Peer review: Reviewer response for version 2

**DOI:** 10.21956/wellcomeopenres.16831.r36187

**Published:** 2019-08-13

**Authors:** Rose McGready

**Affiliations:** 1 Shoklo Malaria Research Unit, Mahidol University, Mae Sot, Thailand

I have reviewed Version 2 and the response to comments have been satisfactorily addressed by the authors. The inclusion of the TRACT manuscripts provided in this version was worth waiting for. This is very important to get indexed and provides good evidence to support a clinical trial on transfusion in children not just for sSA but other parts of the globe e.g. South and South East Asia where severe *P. falciparum* occurs.

I confirm that I have read this submission and believe that I have an appropriate level of expertise to confirm that it is of an acceptable scientific standard.

# Peer review: Reviewer response for version 1

**DOI:** 10.21956/wellcomeopenres.16367.r34837

**Published:** 2019-03-06

**Authors:** Rose McGready

**Affiliations:** 1 Shoklo Malaria Research Unit, Mahidol University, Mae Sot, Thailand

The authors have presented a significant data bundle (8 years, >29,000 children) to understand survival in transfused children when using the thresholds in the WHO guidelines. Other admission characteristics or investigations e.g. malaria, signs of severity; were included in the analysis. The very high burden and mortality from profound and severe anaemia, 10 years ago (2002-2009) in Kilifi Kenya, was presented. The manuscript is well written and a large amount of information (a great deal of clinical work) has been packaged together to provide insight on an important subject.

 Major:

1. The data is nearly 10 years old – can the reason for presenting this particular 2002-2009 cohort be explained?
2. Is there overlap between FEAST study and this large dataset? If yes – that is a bit different to routine hospital admission and treatment.
3. The take away message (as in first sentence of the abstract - conclusion, that states that transfusion does not improve outcome in severe and complicated anaemia) is profound, and contrary to long held belief/practices. Are there important missing data that are unavailable or is there a limitation to the data analysis? This bold statement comes from a stratified analysis. There is sometimes a frustrating amount of missing data e.g. base excess, HIV in Table 3. Is it the missing data that precluded multivariate analysis? Can all/more variables be added to the Table 5 analysis? Does mortality risk change over time (year of admission)?

Minor:

1. Affiliation 2 has two commas after facility.
2. Abstract Background: These first 3 sentences lack cohesion. For the first sentence – not just pediatric transfusion that are challenging ….all transfusion? What is the connection between restrictive transfusion practices (from HIC?) and the next statement about survival, malaria +/- transfusion.
3. Abstract Methods: A retrospective analysis of hospital records of children? Digital records? Paper records?
4. sSA undefined at first use in Conclusion of Abstract.
5. Introduction - Wording problem in this sentence: For example, the 2016 WHO survey found that of 46 countries reporting in the WHO African Region, which are home to approximately 13% of the global population collected a total of about 5.6 million blood donations and accounted for only about 4% of global donations ^2^.
6. What is HDI?
7. Methods 2 ^nd^ paragraph - Wording problem: “…systematically collected on all admission…
8. Discussion – section what new knowledge this study contributes.
9. The later half of the 2 ^nd^ sentence “…receipt of a transfusion MAY NOT be beneficial irrespective of haemoglobin levels including children with PROFOUND malarial anaemia” appears contradictory to the  first ½ of the first sentence of the same paragraph “…for children with malaria there MAY  be a benefit of transfusion for those with PROFOUND anaemia”.
10. It would be useful for the reader to include the sample size of the Canadian and European restrictive transfusion trial (given the sample size data at the start of the paragraph).
11. Malaria has decreased but has HIV has as well?
12. Conclusion, 2 ^nd^ word: ‘poor’ or ‘good’ compliance with current guidelines?

I confirm that I have read this submission and believe that I have an appropriate level of expertise to confirm that it is of an acceptable scientific standard, however I have significant reservations, as outlined above.

# Response

**Published:** 2019-08-05

**Authors:** Kathryn Maitland

**Affiliations:** KEMRI-Wellcome Trust Research Programme, Kenya

Major:

The data is nearly 10 years old – can the reason for presenting this particular 2002-2009 cohort be explained? Is there overlap between FEAST study and this large dataset? If yes – that is a bit different to routine hospital admission and treatment.

**Yes the data sets were assembled to inform the design of the TRACT trial (and justify to funders) - see reference to trial protocol.  The data were collected during a period when malaria transmission in Kilifi  had transitioned from high endemicity to low (its nadir actually), based upon the recent paper published about severe malaria** (ref 1 below) **. FEAST trial only started in 2009 and the numbers included in the trial from Kilifi were relatively small and I do not think this would have influenced the overall findings.**

The take away message (as in first sentence of the abstract - conclusion, that states that

transfusion does not improve outcome in severe and complicated anaemia) is profound, and

contrary to long held belief/practices. Are there important missing data that are unavailable or is there a limitation to the data analysis? This bold statement comes from a stratified analysis. There is sometimes a frustrating amount of missing data e.g. base excess, HIV in Table 3. Is it the missing data that precluded multivariate analysis? Can all/more variables be added to the Table 5 analysis? Does mortality risk change over time (year of admission)?

**We have moderated the sentence to say severe anaemia. The only parameters that were collected routinely were clinical data, haemoglobin, malaria slide and blood cultures. HIV was only recorded on patients following parental assent; thus missingness in this data field is likely to be bias the overall interpretation. Blood gases were not routinely collected and only done in children with signs of severity and at a clinicians discretion.**

Minor:

Affiliation 2 has two commas after facility.

**We have corrected these.**

Abstract Background: These first 3 sentences lack cohesion. For the first sentence – not just

pediatric transfusion that are challenging ….all transfusion? What is the connection between

restrictive transfusion practices (from HIC?) and the next statement about survival, malaria +/-

transfusion.

Abstract Methods: A retrospective analysis of hospital records of children? Digital records? Paper records? sSA undefined at first use in Conclusion of Abstract.

**We have corrected these and rewritten the first 3 sentences to make our points clearer.  The methods of data collection are made clearer.**

Introduction - Wording problem in this sentence: For example, the 2016 WHO survey found that of 46 countries reporting in the WHO African Region, which are home to approximately 13% of the global population collected a total of about 5.6 million blood donations and accounted for only about 4% of global donations. What is HDI? Methods 2 paragraph - Wording problem: “…systematically collected on all admission…

**We have edited these.**

Discussion – section what new knowledge this study contributes.

**We have included this.**

The latter half of the 2 sentence “…receipt of a transfusion MAY NOT be beneficial irrespective of haemoglobin levels including children with PROFOUND malarial anaemia” appears contradictory to the first ½ of the first sentence of the same paragraph “…for children with malaria there MAY be a benefit of transfusion for those with PROFOUND anaemia”.

**Sorry, thank you for pointing out this important typo - we agree that the later sentence is the correct version**.

It would be useful for the reader to include the sample size of the Canadian and European restrictive transfusion trial (given the sample size data at the start of the paragraph).

**We are not sure what a sample size would add for observational data? The Canadian/European cohorts were much lower sample sizes and based on non-inferiority.**

Malaria has decreased but has HIV has as well?

Conclusion, 2 word: ‘poor’ or ‘good’ compliance with current guidelines?

**We don't have good evidence that there have been changes in HIV overtime; we do however for malaria.**

**We have clarified wording in the conclusions.**

1.         Njuguna P, Maitland K, Nyaguara A, et al. Observational study: 27 years of severe malaria surveillance in Kilifi, Kenya. *BMC Med* 2019; **17**(1): 124.

# Peer review: Reviewer response for version 1

**DOI:** 10.21956/wellcomeopenres.16367.r34854

**Published:** 2019-03-06

**Authors:** Hans Ackerman

**Affiliations:** 1 Laboratory of Malaria and Vector Research, Division of Intramural Research, National Institute of Allergy and Infectious Diseases, National Institutes of Health, Rockville, MD, USA

Summary: This is a prospective observational study of consecutive admissions of children age 60 days to 15 years to Kilifi County Hospital during the years 2002-2009. The primary goal of the analysis was to calculate in-hospital mortality within each hemoglobin stratum (e.g., 3.0-3.9 g/dL). Secondary goals were to see if mortality by Hb stratum differed between children with malaria versus children hospitalized for other reasons, and to see if mortality was different between those who received a blood transfusion versus those who did not.

 Strengths of this study include: (1) established data collection practices; (2) routine clinical evaluations including physical assessment, complete blood count, blood culture, HIV testing, and blood film for malaria parasites; (3) consistent method of determining Hb concentration (Coulter counter); and (4) a large sample size with 29,226 included in the analysis. With this wealth of data from a single site over many years, there is the potential to learn more about transfusion practice and transfusion thresholds.

 However, the use of stratification instead of adjustment (e.g., multivariable regression modeling) limits the conclusions that can be drawn from the data. The results could look different if adjustment for disease severity were applied instead of the current approach of stratification and subgroup analysis. Take, for example, the analysis presented in Table 4a. This table shows the case fatality rate for children with malaria stratified by hemoglobin level, comparing those who were transfused against those who were not transfused. When the Hb level was in the range of 5.0-5.9 g/dL, the mortality in the not transfused group was 16/479 (3.3%) and in the transfused group it was 20/178 (11.2%), giving an unadjusted odds ratio for death associated with transfusion of 3.66 (95%CI 1.85-7.24, p < 0.001) - in other words, transfusion appears to be associated with a substantially increased odds of death in children with malaria who have a hemoglobin in the range of 5.0-5.9 g/dL.

 The problem with this analysis is that it doesn't consider all the information available for each patient. We know that WHO guidelines were followed most of the time at Kilifi County Hospital, and that to transfuse a child with malaria whose hemoglobin is in the range of 4.0 - 6.0 g/dL, an additional life-threatening  complication would need to be present to justify the transfusion. These life-threatening complications could include any one of the following: shock, severe dehydration, impaired consciousness, respiratory distress or high parasitemia. The presence of these life-threatening complications in the transfused group (and to a much lesser extent in the not transfused group) could explain the higher mortality observed. To accurately determine the association between transfusion and death, one needs to account for the underlying differences in disease severity that may exist between the transfused and not transfused groups. In addition to the factors listed above, one may need to include base excess, age, nutritional status and possibly other factors that affect the outcome of hospitalization in a multivariable analysis.

 The authors have approached this issue with a subgroup analysis shown in Table 5 where the analysis was limited only to those children who have deep acidotic breathing and/or altered consciousness. While it would appear that transfusion is largely futile, and possibly harmful, in this subgroup of severely ill children, it is hard to interpret the results when other potential determinants of outcome such as age, malaria infection, HIV status, nutritional status, trauma, etc., might not be equally distributed across the transfused and not transfused groups like they would be if this were a randomized controlled trial.

 For these reasons, I would caution the authors not to draw conclusions from the comparisons of crude mortality rates in unadjusted analyses when the underlying disease severity may be very different among those who received transfusions compared to those who did not. I would recommend a statistical approach where the effects of multiple factors and their interactions could be accounted for.

 Overall, the article represents a tremendous data collection effort that has produced a rich dataset with the potential to inform both current practice in Eastern Kenya as well as the design of randomized controlled trials for blood transfusion of children living in sub-Saharan Africa.

 --

 Other comments:

 While there appeared to be very tight adherence to WHO guidelines for Hb < 4 g/dL (912/1134 [80.4%] transfused) and for Hb > 6 (520/24,372 [2.1%] transfused), there was much less adherence to the guidelines when Hb was in the 4-6 g/dL range. In the 4-6 g/dL range, for those who met severity criteria for transfusion, only 567/1564 (36.3%) were transfused, and for those who did not meet severity criteria, 194/746 (26.0%) were still transfused - why do you think adherence was less strict when the hemoglobin was in the 4-6 g/dL range?

 In the discussion, it may be worth emphasizing that while Canadian and European trials in critically ill children have established that a transfusion threshold of 7 g/dL is not worse than a transfusion threshold of 9 g/dL, this is still well above the threshold of 4 (or 6) g/dL recommended worldwide where little trial data is available.

 In reference to the FEAST trial, the fluid bolus volume was up to 60 mL/kg in stratum B of that study (not 40 mL/kg as cited here).

 Regarding baseline characteristics:

- Does mid-upper arm circumference need to be normalized to age?
- Was tachycardia defined using age-specific ranges?
- Was temperature gradient determined by touch or with a thermometer?
- How was coma defined?

--

 Minor comments:

 Figure 2 would benefit from an legend explaining the comparisons being made: each Hb stratum vs the sum of all strata above that one.

 Table 2b was referred to but not present in the pdf version of the article.

*© 2019 Hans Ackerman. This is an open access peer review report distributed under the terms of the Creative Commons Attribution Licence, which permits unrestricted use, distribution, and reproduction in any medium, provided the original work is properly cited. The author(s) is/are employees of the US Government and therefore domestic copyright protection in USA does not apply to this work. The work may be protected under the copyright laws of other jurisdictions when used in those jurisdictions.*

I confirm that I have read this submission and believe that I have an appropriate level of expertise to confirm that it is of an acceptable scientific standard, however I have significant reservations, as outlined above.

# Response

**Published:** 2019-08-05

**Authors:** Kathryn Maitland

**Affiliations:** KEMRI-Wellcome Trust Research Programme, Kenya

Thank you for your very detailed consideration of this manuscript. First,  I want to apologise for my late response to their questions, I have been unusually distracted by other commitments but also timed my response so I could refer to the TRACT trial transfusion manuscripts which are now published and help to address some of the questions raised.  I will respond to the reviewers individual comments.  

Summary: This is a prospective observational study of consecutive admissions of children age 60 days to 15 years to Kilifi County Hospital during the years 2002-2009. The primary goal of the analysis was to calculate in-hospital mortality within each hemoglobin stratum (e.g., 3.0-3.9 g/dL). Secondary goals were to see if mortality by Hb stratum differed between children with malaria versus children hospitalized for other reasons, and to see if mortality was different between those who received a blood transfusion versus those who did not.

Strengths of this study include: (1) established data collection practices; (2) routine clinical evaluations including physical assessment, complete blood count, blood culture, HIV testing, and blood film for malaria parasites; (3) consistent method of determining Hb concentration (Coulter counter); and (4) a large sample size with 29,226 included in the analysis. With this wealth of data from a single site over many years, there is the potential to learn more about transfusion practice and transfusion thresholds.

However, the use of stratification instead of adjustment (e.g., multivariable regression modeling) limits the conclusions that can be drawn from the data. The results could look different if adjustment for disease severity were applied instead of the current approach of stratification and subgroup analysis. Take, for example, the analysis presented in Table 4a. This table shows the case fatality rate for children with malaria stratified by hemoglobin level, comparing those who were transfused against those who were not transfused. When the Hb level was in the range of 5.0-5.9 g/dL, the mortality in the not transfused group was 16/479 (3.3%) and in the transfused group it was 20/178 (11.2%), giving an unadjusted odds ratio for death associated with transfusion of 3.66 (95%CI 1.85-7.24, p < 0.001) - in other words, transfusion appears to be associated with a substantially increased odds of death in children with malaria who have a hemoglobin in the range of 5.0-5.9 g/dL.

The problem with this analysis is that it doesn't consider all the information available for each patient. We know that WHO guidelines were followed most of the time at Kilifi County Hospital, and that to transfuse a child with malaria whose hemoglobin is in the range of 4.0 - 6.0 g/dL, an additional life-threatening complication would need to be present to justify the transfusion. These life-threatening complications could include any one of the following: shock, severe dehydration, impaired consciousness, respiratory distress or high parasitemia. The presence of these life-threatening complications in the transfused group (and to a much lesser extent in the not transfused group) could explain the higher mortality observed. To accurately determine the association between transfusion and death, one needs to account for the underlying differences in disease severity that may exist between the transfused and not transfused groups. In addition to the factors listed above, one may need to include base excess, age, nutritional status and possibly other factors that affect the outcome of hospitalization in a multivariable analysis.

The authors have approached this issue with a subgroup analysis shown in Table 5 where the analysis was limited only to those children who have deep acidotic breathing and/or altered consciousness. While it would appear that transfusion is largely futile, and possibly harmful, in this subgroup of severely ill children, it is hard to interpret the results when other potential determinants of outcome such as age, children, it is hard to interpret the results when other potential determinants of outcome such as age, malaria infection, HIV status, nutritional status, trauma, etc., might not be equally distributed across the transfused and not transfused groups like they would be if this were a randomized controlled trial.

For these reasons, I would caution the authors not to draw conclusions from the comparisons of crude mortality rates in unadjusted analyses when the underlying disease severity may be very different among those who received transfusions compared to those who did not. I would recommend a statistical approach where the effects of multiple factors and their interactions could be accounted for.

Overall, the article represents a tremendous data collection effort that has produced a rich dataset with the potential to inform both current practice in Eastern Kenya as well as the design of randomized controlled trials for blood transfusion of children living in sub-Saharan Africa.

Response:

**We agree that the looking at a Hb range over 1 gram increments is rather crude but the numbers in each of the levels of Hb are large. We were limited in adding in too many other variables to understand why the transfusion vs no transfusion may have had different outcomes as** **the only parameters that were collected routinely were clinical data, haemoglobin, malaria slide and blood cultures. HIV was only recorded on patients following parental assent; thus missingness in this data field is likely to bias the overall interpretation as those refusing the test have been shown to have worse outcome. Blood gases were not routinely collected and only done in children with signs of severity and at a clinicians discretion, thus likely to bias the interpretation if this were to be included in a multivariate analysis. Moreover, the decision to transfuse, especially in those with higher haemoglobin were not routinely collected and thus a major limitation of this paper. The only way to satisfactorily address whether children benifit from transfusion is through a clinical trial which has been conducted in those with haemoglobins below 6 g/dl** **(2). For those with Hb above this level this would require a separate trial, which may be harder to justify given the substantial burden that severe anaemia places on the transfusion services in Africa.**

Other comments:

While there appeared to be very tight adherence to WHO guidelines for Hb < 4 g/dL (912/1134 [80.4%] transfused) and for Hb > 6 (520/24,372 [2.1%] transfused), there was much less adherence to the guidelines when Hb was in the 4-6 g/dL range. In the 4-6 g/dL range, for those who met severity criteria for transfusion, only 567/1564 (36.3%) were transfused, and for those who did not meet severity criteria, 194/746 (26.0%) were still transfused - why do you think adherence was less strict when the hemoglobin was in the 4-6 g/dL range?

Response:

**Yes, we were surprised by the poor compliance in those with Hb 4-6g/dl with current guidelines especially those with signs of severity. Some may have been explained by blood shortages and by the time blood became available either the child had died or recovered- we did not record whether a transfusion was requested and not received- something for future implementation post-TRACT trial. In the TRACT trial we did note that 49% of children with Hb 4-6 g/dl without signs of severity developed severe and complicated  anaemia later in the admission requiring blood transfusion**(3) **. We have now included this into the discussion section of the paper.**

In the discussion, it may be worth emphasizing that while Canadian and European trials in critically ill children have established that a transfusion threshold of 7 g/dL is not worse than a transfusion threshold of 9 g/dL, this is still well above the threshold of 4 (or 6) g/dL recommended worldwide where little trial data is available.

**We have added in some more discussion about this include a review that was done regarding this.**

In reference to the FEAST trial, the fluid bolus volume was up to 60 mL/kg in stratum B of that study (no  40 mL/kg as cited here).

**In the controlled trial (Albumin vs Saline (Bolus) vs Control which is the reference I am referring to only received 20-40 mls/kg only (with 40 mls/kg given only in the last 6 months of the trial, after a protocol amendment) In the stratum with hypotensive shock only few children were enrolled (29 in total) and there was no controls in this stratum and in reality following the protocol amendment which permitted up 60mls/kg very few children actually received 60mls/kg.**

Regarding baseline characteristics: Does mid-upper arm circumference need to be normalized to age?

**This is a standard way of assessing MUAC; zscores are unusual.**

Was tachycardia defined using age-specific ranges? Was temperature gradient determined by touch or with a thermometer? How was coma defined?

**Yes, we have included in the methods and for tachycardia I have reference the FEAST trial.**

Minor comments:

Figure 2 would benefit from a legend explaining the comparisons being made: each Hb stratum vs the sum of all strata above that one. Table 2b was referred to but not present in the pdf version of the article.

**Sorry this was in error it was meant to indicate the legend below Table 4b.**

1.         Njuguna P, Maitland K, Nyaguara A, et al. Observational study: 27 years of severe malaria surveillance in Kilifi, Kenya. *BMC Med* 2019; **17**(1): 124.

2.         Mpoya A, Kiguli S, Olupot-Olupot P, et al. Transfusion and Treatment of severe anaemia in African children (TRACT): a study protocol for a randomised controlled trial. *Trials* 2015; **16**(1): 593.

3.         Maitland K, Kiguli S, Olupot-Olupot P, et al. Immediate Transfusion in African Children with Uncomplicated Severe Anemia. *The New England journal of medicine* 2019; **381**(5): 407-19.
